# A Differential Threshold of Breakfast, Caffeine and Food Groups May Be Impacting Mental Well-Being in Young Adults: The Mediation Effect of Exercise

**DOI:** 10.3389/fnut.2021.676604

**Published:** 2021-07-05

**Authors:** Lina Begdache, Hamed Kianmehr, Helen Najjar, Dylan Witt, Nasim S. Sabounchi

**Affiliations:** ^1^Health and Wellness Studies Department, Binghamton University, Binghamton, NY, United States; ^2^Department of Pharmaceutical Outcomes and Policy, College of Pharmacy, University of Florida, Gainesville, FL, United States; ^3^Department of Biomedical Engineering, Watson College of Engineering, Binghamton University, Binghamton, NY, United States; ^4^Department of Biological Sciences, Binghamton University, Binghamton, NY, United States; ^5^Department of Health Policy and Management, Center for Systems and Community Design, CUNY Graduate School of Public Health & Health Policy, New York, NY, United States

**Keywords:** dietary patterns, food groups, exercise, mental health, gender, young adults, caffeine, mediation

## Abstract

Diet and exercise are known to influence mental health. However, the interaction between diet, dietary practices, and exercise and its impact on the mood of young adults (YA) is poorly understood. YA are inherently at risk for mental distress. They tend to consume a low-quality diet and are generally active. The purpose of the study was to assess these relationships through validating causal loop diagrams (CLD) that describe these connections by using a system dynamic (SD) modeling methodology. Adults 18–29 years were invited to complete the Food-Mood questionnaire. The anonymous questionnaire link was distributed to several institutional listservs and via several social media platforms targeting young adults. A multi-level analysis, including machine learning techniques, was used to assess these relationships. The key findings were then built into gender based CLD, which suggest that a differential repertoire may be needed to optimize diet quality, exercise, and mental well-being. Additionally, a potential net threshold for dietary factors and exercise may be needed to achieve mental well-being in young adults. Moreover, our findings suggest that exercise may boost the enhancing effect of food groups on mental well-being and may lessen the negative impact of dietary impediments of mental well-being.

## Introduction

Several lines of evidence indicate that diet and exercise have an impact on mental health. However, little is known about their influence on the mood of young adults (YA) as a specific sub-population. Most studies that confirmed the relationship between poor quality diet and mental distress in adults included a wide range of ages (1–4). There is scarcity in research performed exclusively on young adults, not to mention the paucity of this research characterized by gender. There is an increase evidence that morphological differences exist in the gender brain (5–8), which may modulate dietary needs to support mental well-being. An interventional pilot study reported that a 10-day Mediterranean diet significantly improved mood in young females (9). A randomized controlled study suggested that dietary interventions significantly reduced depressive symptoms in young adults. There are several reports that addressed the role of exercise on the mood of young adults. A scoping review that included thirty publications on the effect of physical activity and exercise on mental health in young adults reported that interventions of different intensities may reduce depression and anxiety symptoms (10). Even an acute vigorous-intensity aerobic exercise was reported to significantly reduce anxiety scores in young adults (11). However, the high consumption of caffeine by young adults through energy drinks and caffeinated beverages may be increasing their risk for mental distress. Caffeine is a psychoactive and addictive substance that was reported to modify the neuroendocrine stress response in adolescents and young adults leading to an increased vulnerability to mental health disorders (12).

Therefore, YA are inherently at a higher risk for mental distress with a tendency for impulsivity (13, 14), more likely to consume a low-quality diet (15) and tend to be generally active (16), which make them an attractive cohort for studying the interrelation between mood, diet and exercise. YA have a poor control over their emotions, which is a characteristic attributed to the underdeveloped functional regions of the prefrontal cortex (PFC) (17). Therefore, a high-quality diet and frequent exercise contribute substantially to brain development (18, 19). Since the young brain completes its full development by mid-late 20s, optimizing diet and exercise is necessary to improve mental health in young adults. In fact, PFC development is a critical temporal window of neuroplasticity that involves neural reorganization and cognitive functions development. These processes are typically orchestrated by a robust interaction between genetic and environmental factors. PFC regulates emotions and thought processes through a network of brain connectivity that synchronizes the release of several neurotransmitters involved in mood regulation.

Therefore, positive influencers of neuroplasticity during this vital stage of development may support a gender-based PFC development with a potential long-lasting effect. Regular exercise has been linked to improvements in cognitive functions, memory and mood (19–21). Biochemically, exercise boosts the release of several growth and neurotrophic factors that support brain development and homeostasis (22). Cardiovascular and strength exercise training induces considerable neurochemical and structural modifications in the brain. Specifically, the brain derived neurotrophic factor (BDNF) stimulates the proliferation of stem cells into functional neurons and supports neuroplasticity (23). Vascular endothelial growth factor (VEGF) promotes angiogenesis to support oxygen perfusion and nutrient delivery, which are critical for the developing brain (24). Insulin-like growth factor (IGF-1) is a key player in brain development as it supports neuroplasticity, neuroprotection and neural repair (25). In essence, exercise optimizes brain development in young adults, which supports mental well-being. Additionally, imaging studies revealed that there is a difference in brain morphology between men and women (5). In addition, there is a gender difference in brain connectome (26), which explains the several differences in behavioral and emotional processes between men and women. In fact, women are twice as likely to experience mental distress, with longer episodes and higher risk for relapse (27).

**Figure 1:**
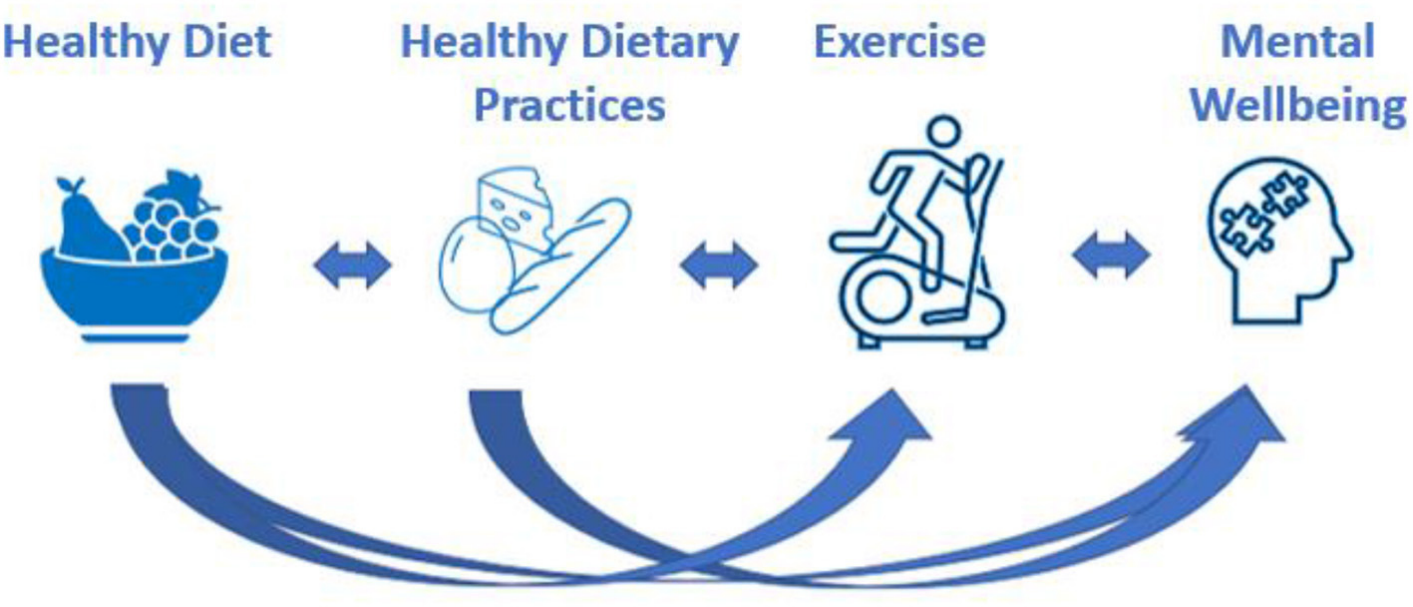
Graphical representation of the hypothetical model. The arrows represent positive impact. Diet impacts exercise and mental well-being. Dietary practices impact exercise and mental well-being. Each component of the model has a reversal relationship with its neighboring component resulting in mental well-being improving diet quality and dietary practices.

Several studies reported on the role of diet and exercise in mental well-being; however, the effect of dietary patterns, dietary practices, and exercise frequency on mental distress in young adults is poorly understood. Based on a qualitative and hypothetical system dynamics (SD) model identifying causal and feedback loop structures published by our team, eating healthy and/or adopting healthy practices in young adults boost exercise frequency (28). According to this model, these positive behaviors are likely to modify brain chemistry and act as positive reinforcers to further support a healthy lifestyle. Therefore, this positive feedback loop is hypothesized to promote mental well-being (Figure 1). However, these results were based on a backward regression analysis of data collected from young adults, and the model has not been verified in the literature. We wish to take this study one step further and investigate these relationships based on gender and validate the model. To test and validate causal loop diagrams (CLD), partial testing of loop segments using newly generated data and several statistical methods are needed (29). Therefore, the aim of this study was to examine the different segments of the CLD by using machine learning and other statistical methods. Another aim was to investigate the gender-based internal processes that interlace the associations between diet, dietary practices, exercise, and mental well-being, which is another area in need of further research. Validating this hypothetical model in young men and women and recognizing the individual connections between food groups, exercise, and mental distress could serve as a guide for customizing dietary plans to promote exercise and improve mental well-being in young adults.

## Method

### Participants

The study protocol was reviewed and approved by the institutional Internal Review Board. This study is part of a larger study designed to explore dietary patterns and mental distress, which aims to investigate the impact of food groups on mental distress based on gender and age groups. The study design involves repeated cross-sectional design and collected data from an international sample of adults 18 years and older. However, for the purpose of the current study, data from 18 to 29 years were only considered. Data collection from young adults also included distribution of the questionnaire link to several institutional listservs and via several social media platforms targeting current college students and graduates in the workforce. Participants consented to the study by agreeing to access the survey. Data were examined for back-to-back entries using date and time stamps to identify potential double entries. A minimum sample size with 95% confidence and 5% margin of error was set at 384 samples for each gender using the online tool, https://www.surveysystem.com/sscalc.htm#one. However, to boost the study power of the machine learning analyses, collection of responses went beyond the minimum sample size needed. Data collection was performed over a 3-year period and at different intervals to account for the change in season as well as to diversify the target population. As an added measure, a duplicate function was performed to remove double entries. A sensitivity analysis was also performed to determine the extent to which suspicious entries differ from the remaining study responses.

### Materials

Dietary and nutrient consumption patterns were assessed by using the Food-Mood Questionnaire (FMQ) (30). FMQ is a validated instrument that has been previously used in the nutritional neuroscience field (28, 31). Briefly, FMQ evaluates weekly servings of food groups known to influence brain function and chemistry. These food groups include whole grain, fruits, dark green leafy vegetables, meat (white and red), beans and legumes, nuts, dairy, fish, and high glycemic index (HGI) foods. Frequency of breakfast consumption and exercise, use of multivitamins and fish oil supplements, consumption of fast-food and caffeinated beverages was also assessed. FMQ includes a question on frequency of exercise for at least 20 min, which was based on the evidence from the literature that exercising at least 20 min a day, regardless of the type of exercise, improves mood (32–34). Additionally, FMQ usefulness includes a concise classification of dietary patterns within the dataset (30). FMQ is a 5-subscale item with an internal consistency, as reflected by Cronbach's alpha values >0.70 for all sub-scales. FMQ is a reliable tool (Intraclass Class Coefficient 0.619–0.884; *P* < 0.01; CI 95%), which has an external validity as well (30). Demographic questions included gender, age-groups (18–29, 30–39, 40–49, 50 and above), region of residence, highest education achieved (High school, 2–4 years of college AA, BS, BA), and dietary patterns followed. FMQ questions use a 6-point Likert scale and include six questions on mental distress adopted from Kessler-6 Psychological Distress Scale (K-6) (35). The K-6 scale is a quantifier of a spectrum of mood and its questions originate from Item Response Theory. K-6 has consistent psychometric properties across major socio-demographic sub-samples and strongly discriminates between community cases and non-cases of DSM-IV/SCID (36). Therefore, the total sum of K-6 was used to assess mental distress.

### Classification of Dietary Patterns

The Healthy Dietary Pattern was classified based on the recommendations by the Dietary Guidelines for Americans 2015–2020 (37), which include a spectrum of nutrient-dense food such as fruits, vegetables, whole grains, beans, nuts lean meat and low-fat dairy. The Unhealthy Dietary Pattern was classified based on the USDA published results of the 1,994 Continuing Survey of Food Intakes by Individuals (CSFII), describing the Standard American Diet (38), which includes dairy, meat, high glycemic index food, fast food and excludes fruits, vegetables, legumes and fish consumption. Supplement Pattern was established around the clustering of multivitamin and fish oil supplements use based on the FMQ validation study (30).

### Data Partitioning

Adults between 18 and 29 years were categorized as young adults (YA) as suggested by Somerville (39). To classify the degree of mental distress among participants, K-6 sum scores were categorized into three levels: Low mental distress (0–7), moderate (8–12), and high (13 and above) (40), which is commonly used in the literature (36).

### Statistical Analysis

#### Data Pre-processing

Several missing data values were encountered in the dataset. To avoid loss of valuable information, a Multiple Imputation by Chained Equation (MICE) was performed. MICE treats every variable with a missing value as a dependent variable in a regression analysis, while the remaining variables are considered predictors. Several cycles were performed and followed by a “predictive mean matching” (PMM) that produces arbitrary draws from the predictive fitted models. These random draws become the imputed values. The MICE R package was used to impute the missing data (24).

#### Methods and Rationale of the Study Design

**Figure 2:**
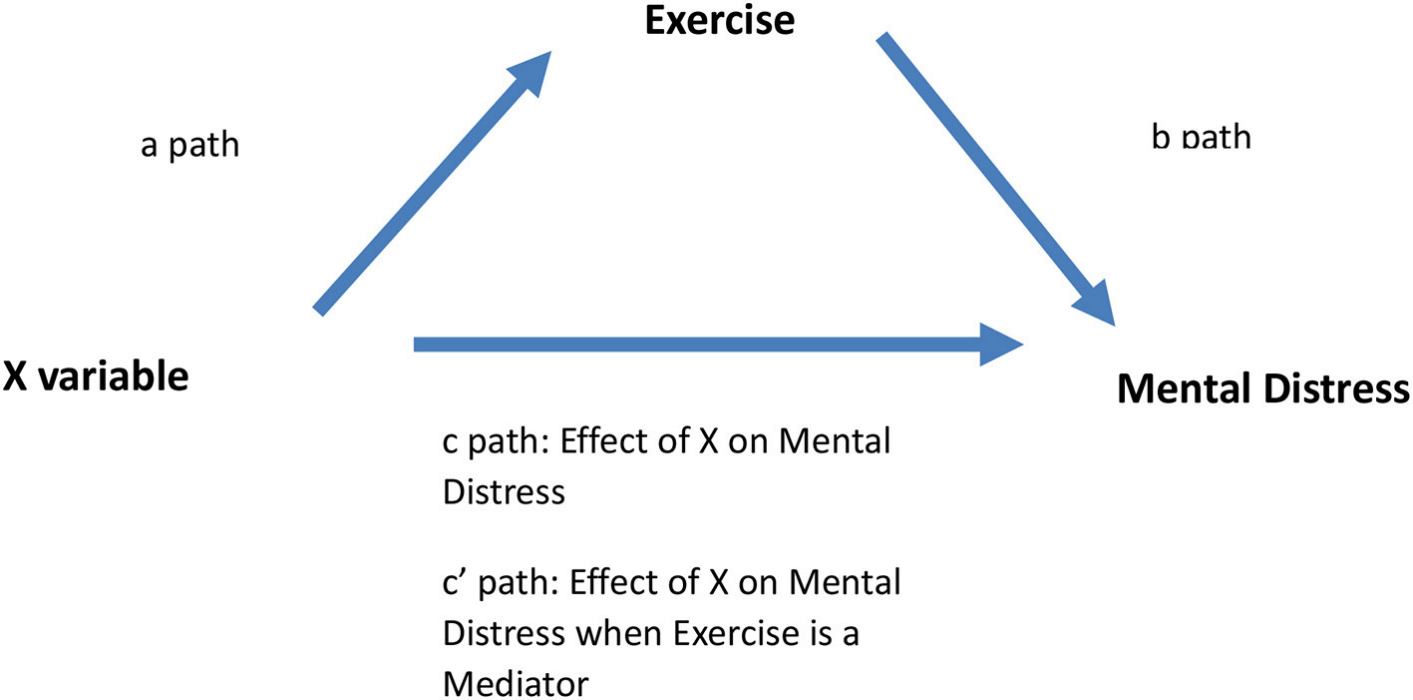
The 3 paths of the mediation model analyzed.

**Figure 3:**
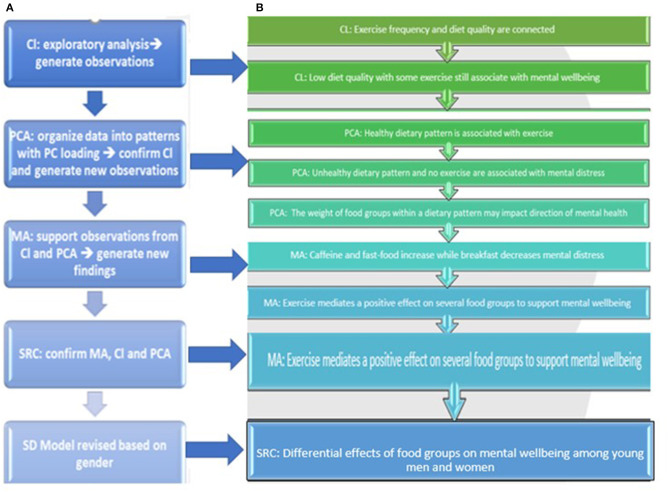
**(A,B)** Research design and rationale based on sequence of the results. Cl: cluster analysis; PCA; principal component analysis; MA, mediation analysis; SRC, Spearmen's rho correlation; SD, system dynamics.

The first step was to classify the dataset into clusters to draw inferences from unidentified patterns within a dataset and with no prior labeling. K-means is an unsupervised machine learning technique that detects hidden patterns without human intervention. Next, we used Principal Component Analysis (PCA) to reduce the dimensionality of the data and identify patterns within the dataset. K-means clustering and PCA are powerful methods for visualizing high dimensional data. The third step was to perform a mediation regression analysis (MA) to model the relationships between different variables and assess the role of exercise as a mediator (Figure 2) to explain the observations generated from the previous two analyses. A mediation model identifies the relationship between independent and dependent variables and explains the potential effect of a mediator. The fourth step was to perform a two-tail correlational study to verify the mediation analysis and investigate the individual causal relationships between variables that constitute the different segments of the CLD. In drawing the CLD, we adopt a system dynamics methodology to identify causal links and corresponding feedback loops. A revision of the CLD comprising the hypothetical SD model was followed based on the new findings (Figure 3A).

#### Cluster Analysis

K-means cluster analysis was used as an exploratory study to produce dietary, exercise and mental distress clusters. All variables were standardized into z-scores to give an equal weight and minimize the influence of the outliers. For a given set of observations, K-means clustering aims to group the observations into a pre-specified number of k distinctive, non-overlapping clusters. K-means clustering algorithm uses an iterative enhancement method, which selects data points based on the Euclidean distances between its corresponding centroids. The main idea behind the K-means algorithm is to minimize variations within each cluster. Five steps were followed to identify the clusters. First, k-numbers of the clusters were assigned based on Reynolds et al. suggestions (41). Second, k random numbers were selected from the dataset to establish the initial points. Third, each datapoint was assigned to its closest centroid based on the Euclidean distance. Fourth, the cluster's centroid was recalculated via the value of all data points in the determined cluster. Finally, steps 3 and 4 were repeated until no more changes in the centroid were detected. Participants' characteristics across the dietary clusters were explored using Chi-square analysis. Cluster analysis was performed using R software (Version 1.3.1073).

$$\begin{array}{l} \overset{k}{\underset{k=1}{\sum }}W\left(C_{k}\right)=\overset{k}{\underset{k=1}{\sum }}\underset{x_{i\in C_{k}}}{\sum }{\left(x_{i}-\mu_{k}\right)}^{2} \end{array}$$

Where:

*x*_*i*_ is a datapoint for the *cluster k*

μ_*k*_ is the average of the datapoints in the *cluster k*.

#### Principal Component Analysis

PCA analysis was used to identify the different dietary, exercise and mental distress patterns among respondents. Data were stratified by gender and further by principal components (PC). Sampling adequacy and inter-correlation of variables were assessed using Kaiser-Meyer-Olkin (KMO) test and Bartlett's test of sphericity, respectively. The eigenvalue >1.0 criterion was used to determine the number of PCs retained. The optimal number of components are the ones that capture the highest amount of variance in the dataset. Additionally, the number of PCs selected was confirmed by visually examining the scree plot. Using varimax rotation, PCs were orthogonally rotated to simplify and enhance their interpretability (42). Variables with loading of ≥ 0.2 were considered significant contributors to the patterns and were included in the PC solution (43). Positive and negative loadings suggest direct and inverse relationships with the PCs, respectively. Components were classified into Healthy diet, Unhealthy diet and Supplements use PCs. One-way ANOVA followed by Bonferroni *post-hoc* test was used to compare the means of the dietary patterns.

#### Mediation Analysis

The nine food groups as well as exercise, eating breakfast and use of multivitamins and fish oil supplements are continuous variables that were fed into PROCESS Macro version 3.0, model four (44). To correct for any bias, a 5,000-bootstrapping sampling was set with a 95% confidence interval. The indirect effect is significant, which means there is a mediation effect, if CI does not include zero. Pathway “a” determined the regression coefficients for the effect of the independent variable (X) on exercise. Pathway “b” examined the association between exercise and mental distress. Pathway “c” measured the actual effect of X on mental distress, while pathway “c” estimated the mediation effect (or indirect effect) of exercise on X in relation to mental distress.

#### Spearman's Rho Coefficient Analysis

Data normality was assessed using Shapiro–Wilk and Kolmogorov–Smirnov tests, which suggested that the data deviate from a normal distribution. Consequently, a two-tailed Spearman's rank correlation coefficient Rho, a non-parametric measure of rank correlation, was used to assess the monotonic relationship between the variables of interest. PCA, mediation and correlational analyses were performed using SPSS version 25.0.

## Results

A total of 1,804 records were analyzed from young adults (18–29 years); 644 were from young men, 1,160 were from young women. As an estimate, responses came from over 20 US colleges, and some international responses were captured through the social media platforms.

### Cluster Analysis

The cluster analysis identified 3 dietary patterns (DP) and 2 DP for young men and women, respectively. Characteristics of participants based on cluster classification are presented in Table 1. Using chi-square analysis, the significant variables in Cluster 1 for men included breakfast, fish and supplements use pattern (vitamins and fish oil), which represent a healthy dietary practice. Cluster 2 encompassed a spectrum of nutrient-dense food groups, which represents a healthy dietary pattern. Cluster 3 consists of fast-food, which represents an unhealthy dietary pattern. As for women, Cluster 1 comprised fast-food and meat, which represent an unhealthy dietary pattern. Cluster 2 included a spectrum of nutrient-dense food groups, which represents a healthy dietary pattern (Table 2). Based on the Chi-square analysis, healthy dietary practices and patterns were associated with higher frequency of exercise and lower K-6 scores. Conversely, unhealthy dietary patterns were associated with lower frequency of exercise and higher K-6 scores.

**Table 1:** Participants characteristics of men and women according to dietary patterns identified by the cluster analysis^*^.

		**Men (*****n*** **=** **644)**			***P*-value**	**Women (*****n*** **=** **1,160)**		***P*-value**
		**Healthy dietary practice**	**Healthy dietary pattern**	**Unhealthy dietary pattern**		**Unhealthy dietary pattern**	**Healthy dietary pattern**	
	Less than high school and high school	39	91	123	0.00^**^	296	178	0.17
Education	2 or 4 years of college degree	74	80	148		329	250	
	Master's degree	7	36	24		45	41	
	Doctoral/other professional degree	1	10	11		12	9	
Exercise	None	8	38	83	0.00^**^	170	57	0.00^**^
	1 and 2 times	28	48	102		256	119	
	3 and 4 times	41	50	76		178	182	
	More than 4 times	44	81	45		78	120	
Mental distress	Low (0–7)	74	122	163	0.02^**^	329	278	0.00^**^
	Moderate (8–12)	27	71	80		198	128	
	Severe (13–29)	20	24	63		155	72	

* *All tests are Chi-Square analysis*.

** *P-values < 0.05 were considered significant*.

**Table 2:** Means and standard deviations of food groups by dietary patterns, clusters of shaded values represent the variables with the highest frequencies between clusters.

**Variables**		**Men**			**Women**	
		**Cluster**			**Cluster**	
		**Healthy dietary practice**	**Healthy dietary pattern**	**Unhealthy dietary pattern**	**Unhealthy dietary pattern**	**Healthy dietary pattern**
	121	217	306	682	478
Breakfast	Mean	0.33884	0.257412	−0.2887	−0.26937	0.377565
	Stdev	0.818844	0.914713	1.030566	1.069007	0.746369
Whole Grain	Mean	0.171488	0.655982	−0.48348	−0.44165	0.619044
	Stdev	0.902278	0.945307	0.787114	0.823233	0.892245
Dairy	Mean	0.271411	0.289478	−0.2847	−0.15433	0.216322
	Stdev	0.934008	0.98962	0.950559	0.938253	1.043829
Caffeine	Mean	0.309517	0.177955	−0.22702	−0.24296	0.340541
	Stdev	0.939634	1.040418	0.942736	0.966552	0.946155
Fruits	Mean	0.102298	0.616993	−0.43324	−0.47688	0.668422
	Stdev	0.98806	0.936958	0.809883	0.785888	0.878194
Nuts	Mean	0.306594	0.384391	−0.35843	−0.45203	0.633593
	Stdev	1.046821	1.069514	0.788029	0.693086	1.02058
HGI food	Mean	0.072918	0.454513	−0.31826	−0.01951	0.027343
	Stdev	1.012968	0.892965	0.943424	0.979314	1.028695
Meat	Mean	0.12797	0.182422	−0.16371	0.062427	−0.0875
	Stdev	1.019616	0.970065	0.98761	0.932704	1.082285
DGLV	Mean	0.22002	0.726219	−0.54624	−0.44458	0.623153
	Stdev	0.963755	0.777801	0.792839	0.901894	0.772669
Beans	Mean	0.019855	0.618207	−0.40397	−0.40277	0.564548
	Stdev	0.924096	1.086294	0.730743	0.773698	1.008856
Fish	Mean	0.389198	0.303935	−0.33691	−0.14982	0.209993
	Stdev	1.048985	1.075948	0.800095	0.91208	1.078023
Fast-food	Mean	−0.07252	−0.17357	0.137824	0.144997	−0.20324
	Stdev	1.044019	0.988799	0.973413	1.018538	0.937375
Multivitamin	Mean	1.737071	−0.469	−0.33216	−0.15565	0.218172
	Stdev	0.716212	0.396019	0.606169	0.909323	1.078501
Fish oil	Mean	1.108681	−0.23623	−0.2525	−0.09111	0.127707
	Stdev	1.914973	0.291751	0.276075	0.764868	1.247427

*DGLV, dark green leafy vegetables*.

### PCA

Principal component analysis revealed additional interesting findings. There were three principal components (PCs) identified for men and women with a total variance of 34.53 and 36.68%, respectively. For men, PC 1 uncovered strong components representative of a healthy dietary pattern (fruits, leafy vegetables, nuts, beans, whole grain), and exercise with a likelihood of breakfast, fish and caffeine consumption and abstinence from eating fast-food. This PC explained 16.58% of the variance. PC2 had strong positive loading for dairy, caffeine, fast-food and mental distress. Men in this PC are more likely to consume meat and HGI food, and not exercise. This PC explained 10.27% of the total variance. PC 3 reflected a supplements pattern with strong loadings for multivitamin and fish oil. Men in this PC are likely to exercise, eat meat, breakfast, and fish and not experience mental distress. This PC explained 8.89% of the total variance.

For women, PCA produced three distinct PCs similar to the PCs in men with slight variations. PC 1, a healthy dietary pattern, had strong loadings for nuts, fruits, beans, dark green leafy vegetables, whole grain, and exercise. Young women in this PC are more likely to eat breakfast, consume caffeine and avoid fast food and meat. This PC explained 17.26% of the total variance. PC 2, an unhealthy dietary pattern, had strong loadings for dairy, caffeine, fast-food, mental distress. Young women following this pattern are likely to eat meat, HGI food, fruits and are less likely to exercise. This PC explained 10.68 % of the total variance. PC 3 reflected a supplement use pattern with a strong loading for multivitamins and fish oil supplements. Young women in this PC are likely to exercise, eat breakfast, meat and fish and experience mental well-being. This PC explained 8.73 % of the total variance (Table 3).

**Table 3:** PCA analysis on data stratified by gender with varimax rotation.

	**Men**				**Women**		
	**Healthy diet**	**Unhealthy diet**	**Dietary practices**		**Healthy diet**	**Unhealthy diet**	**Dietary practices**
Eigenvalue	2.763	1.650	1.307	Eigenvalue	2.865	1.704	1.300
Variance explained	16.581	10.276	8.890	Variance explained	17.265	10.679	8.734
Fruits	0.669			Nuts	0.716		
DGLV	0.666			Fruits	0.679	0.217	
Nuts	0.661			Beans	0.654		
Beans	0.636			DGLV	0.631		
Whole Grain	0.55			Whole Grain	0.578		
Exercise	0.407	−0.246	0.356	Exercise	0.445	−0.295	0.313
Breakfast	0.356		0.277	Breakfast	0.333		0.306
Fish	0.262		0.218	Dairy		0.669	
Dairy		0.607		Caffeine	0.246	0.65	
Caffeine	0.207	0.6		Fast foods	−0.332	0.489	
Fast foods	−0.333	0.524		Mental distress		0.46	−0.336
Mental distress		0.444	−0.325	HGI		0.271	−0.26
Meat		0.378	0.318	Multivitamin			0.564
HGI food		0.358		Fish oil			0.557
Multivitamin			0.668	Meat	−0.251	0.302	0.457
Fish oil			0.66	Fish			0.289

### Mediation Analysis (c vs. c′ Path)

Using PROCESS Macro, the effect of all nine food groups, breakfast, caffeine, multivitamin and fish oil use on mental distress were explored individually (as X variables) in men and women. The mediation analysis produced novel and interesting findings. Among men, there is a significant association between caffeine, fast food and mental distress. However, exercise significantly reduced the negative effect of caffeine and fast food on mental distress. There was a significant and inverse relationship between breakfast and mental stress; and exercise significantly improved the positive effect of breakfast on mental well-being. Exercise, as a mediator, also generated novel inverse associations between food groups such as whole grain, dairy, fruits, nuts, meat, dark green leafy vegetables, beans, multivitamin supplements, and mental distress. There was no significant effect of fish oil supplements on mental health with or without exercise.

Among women, there was a significant association between caffeine, fast food, and dairy with mental distress. There was a significant and inverse relationship between breakfast, dark green leafy vegetables, and mental stress. Exercise significantly reduced the negative effect of caffeine, fast food, dairy and HGI food on mental distress. Exercise significantly improved the positive effect of breakfast and dark green leafy vegetables on mental well-being. Exercise, as a mediator, also generated novel inverse associations between food groups such as whole grain, fruits, nuts, beans, fish, multivitamin supplements, and mental distress. Exercise did not have any significant effect on meat and fish oil supplements regarding mental well-being in young women (Table 4).

**Table 4:** Mediation regression analysis results based on gender stratification.

**Men**				**Women**			
**Independent**	**Direct effect of X on Mental distress**	**Association between X and Mediator**	**Mediation or Indirect Effect**	**Independent**	**Direct effect of X on Mental distress**	**Association between X and Mediator**	**Mediation or Indirect Effect**
**Variables (X)**	**C path (SE) P**	**A path (SE) P**	**C′** **path (SE) CI**	**Variables (X)**	**C path (SE) P**	**A path (SE) P**	**C′** **path (SE) CI**
Caffeine	0.1379 0.0390 0.0004	−0.05670.03940.1504	0.0092 0.0069 [0.0038_0.0236]	Caffeine	0.1300 0.0162 <0.001	−0.06640.02970.0256	0.0127 0.0062 [0.0017_0.0249]
Breakfast	−0.1350 0.0406 0.0009	0.12910.03850.0008	−0.0196 0.0080 [−0.0373_−0.0061]	Breakfast	−0.1782 0.301 <0.001	0.17990.0301 <0.001	−0.0302 0.0074 [−0.0459_−0.0170]
Whole grain	−0.0065 0.0380 0.8649	0.14070.04090.0006	−0.0237 0.087 [−0.0422_−0.0082]	Wholegrain	−0.0118 0.0254 0.7022	0.17870.0297 <0.001	−0.0354 0.0079 [−0.0519_−0.0208]
Dairy	−0.0377 0.0402 0.3482	0.12870.03960.0012	−0.0212 0.0084 [−0.0391_−0.0069]	Dairy	0.0654 0.0289 0.0237	−0.00910.03020.7641	0.0018 0.0238 [−0.0101_0.139]
Fruits	0.0522 0.0400 0.1925	0.16510.0396 <0.001	−0.0294 0.0096 [−0.0509_−0.0130]	Fruits	−0.0101 0.0252 0.7291	0.21620.0289 <0.001	−0.0428 0.0088 [−0.0612_−0.0267]
Nuts	0.0141 0.0397 0.7106	0.12170.03960.0022	−0.0208 0.0084 [−0.0390_−0.0603]	Nuts	−0.0221 0.0295 0.4532	0.22890.0285 <0.001	−0.0446 0.0087 [−0.0630_−0.0287]
HGI food	0.0710 0.0387 0.0740	0.06160.04070.1309	−0.0107 0.0076 [−0.027_−0.0027]	HGI food	0.0565 0.0292 0.0532	−0.07810.03080.0114	0.0153 0.0031 [0.0031–0.0287]
Meat	0.0308 0.0390 0.4295	0.11450.03980.0041	−0.0198 0.0082 [−0.0378_−0.0061]	Meat	−0.002 0 0.0284 0.9436	−0.02520.02870.4022	0.0050 0.0061 [−0.0067_0.0175]
DGLV	−0.0127 0.0414 0.7592	0.26250.0389 <0.001	−0.0142 0.0078 [−0.0308_−0.0001]	DGLV	−0.0919 0.0279 0.0032	0.32170.0275 <0.001	−0.0548 0.0112 [−0.0784_−0.0343]
Beans	0.02281 0.0384 0.4648	0.08200.04030.0403	−0.0404 0.0085 [−0.0580_−0.0250]	Beans	−0.0274 0.0310 0.3767	0.20800.0280 <0.001	−0.0404 0.0085 [−0.0580_−0.0250]
Fish	0.0746 0.0416 0.0735	0.15310.0366 <0.001	−0.0277 0.0087 [−0.0462_−0.0124]	Fish	−0.0427 0.0286 0.1359	0.07560.02960.0107	−0.0149 0.0063 [−0.0278_−0.0034]
Fast Food	0.1645 0.0410 0.0001	−0.22430.0387 <0.001	0.0297 0.0102 [0.0115–0.0510]	Fast Food	0.1883 0.0292 <0.001	−0.26430.0283 <0.001	0.0330 0.0076 [0.0195–0.0491]
MV	−0.0451 0.0372 0.2258	0.11980.03520.007	−0.0196 0.0073 [−0.0359_−0.0070]	MV	.0.0483 0.0180 0.0848	0.16150.0238 <0.001	−0.0310 0.0075 [−0.0468_−0.0176]
Fish Oil	0.0518 0.0434 0.2329	−0.01950.04000.6264	−0.0066 0.0059 [−0.0187_0.0045]	Fish Oil	−0.0048 0.0315 0.8778	0.03310.02850.2457	−0.0066 0.0059 [−0.0187_0.0045]
B path = −0.1682 SE= 0.0386 *P* < 0.001				B path = −0.1922 SE= 0.0291 *P* < 0.001			

*DGLV, dark green leafy vegetables; HGI, high glycemic index; MV, multivitamin*.

### Mediation Analysis (a Path)

Among men, factors that strongly associated with exercise include breakfast, whole grain, fruits, dark green leafy vegetables, and fish. Other significant factors include dairy, meat, beans, multivitamin supplements. Fast food had a strong inverse relation with exercise. Caffeine, HGI food and fish oil supplements were not significantly associated with exercise. In women, caffeine, HGI food and fast food have an inverse association with exercise. Factors that strongly correlated with exercise include breakfast, whole grain, fruits, nuts, dark green leafy vegetables, beans, multivitamin supplements, and fish. Dairy, meat, and fish oil supplements were not significantly associated with exercise (Table 4).

### Spearman's Rho Correlation

Results from Spearman's Rho correlation analysis are presented in Tables 5, 6. Men and women exhibited common and differential associations between food groups, exercise, breakfast eating, supplements use, and mental distress. Factors that strongly associated with exercise in men, but not in women, are dairy (*r* = 0.132, *p* < 0.01) and meat (*r* = 0.143, *p* < 0.01). No differential factors were observed for mental distress. For women, factors that significantly associated with exercise that were not significant in men are caffeine (*r*= −0.067, *p* < 0.05), HGI food (*r* = −0.075, *p* < 0.01), and beans (*r* = 0.190 *p* < 0.01). For mental distress, whole grain (*r* = −0.049, *p* < 0.05), dairy (*r* = *0*.058, *p* < 0.05), HGI food (*r* = 0.077, *p* < 0.01), dark green leafy vegetables (*r* = −0.145, *p* < 0.01), beans (*r* = −0.065 *p* < 0.05), fish (*r* = −0.051, *p* < 0.05 and multivitamins (*r* = −0.069, *p* < 0.01).

**Table 5:** Spearman's rho correlational analysis results for men.

**Men**	**Exercise**	**Mental distress**	**Breakfast**	**Whole grain**	**Dairy**	**Caffeine**	**Fruits**	**Nuts**	**HGI Food**	**Meat**	**DGLV**	**Beans**	**Fish**	**Fast food**	**MV**	**Fish oil**
Exercise	1.000	–0.165^**^	0.126^**^	0.146^**^	0.132^**^	−0.055	0.166^**^	0.095^*^	0.074	0.143^**^	0.269^**^	0.076	0.149^**^	–0.229^**^	0.078^*^	−0.025
Mental distress		1.000	–0.154^**^	−0.009	−0.057	0.149^**^	0.030	0.008	0.064	0.011	−0.047	0.019	0.036	0.198^**^	−0.039	0.050
Break-fast			1.000	0.173^**^	0.122^**^	0.010	0.143^**^	0.075	0.122^**^	−0.037	0.168^**^	0.137^**^	0.106^**^	–0.142^**^	0.144^**^	−0.032
Whole grain				1.000	0.203^**^	0.120^**^	0.210^**^	0.173^**^	0.124^**^	0.026	0.243^**^	0.157^**^	0.113^**^	−0.018	0.061	−0.064
Dairy					1.000	0.216^**^	0.217^**^	0.161^**^	0.164^**^	0.170^**^	0.075	−0.020	0.091^*^	−0.003	0.014	0.002
Caffeine						1.000	0.139^**^	0.137^**^	0.077	0.018	0.018	0.068	0.031	0.087^*^	0.118^**^	−0.005
Fruits							1.000	0.299^**^	0.195^**^	0.008	0.346^**^	0.223^**^	0.144^**^	–0.105^**^	0.009	−0.038
Nuts								1.000	0.083^*^	−0.017	0.218^**^	0.238^**^	0.182^**^	−0.061	0.092^*^	−0.071
HGIfood									1.000	0.244^**^	0.262^**^	0.202^**^	0.105^**^	0.105^**^	−0.012	0.049
Meat										1.000	0.180^**^	0.006	0.142^**^	0.141^**^	−0.054	0.116^**^
DGLV											1.000	0.367^**^	0.251^**^	–0.134^**^	0.035	−0.037
Beans												1.000	0.250^**^	−0.064	0.043	−0.034
Fish													1.000	−0.042	0.085^*^	0.009
Fast foods														1.000	0.039	0.072
MV															1.000	−0.050
Fish oil																1.000

*DGLV, dark green leafy vegetables; HGI, high glycemic index; MV, multivitamin. Bold signifies statistical significance*.

* *p < 0.05*;

** *p < 0.01*.

**Table 6:** Spearman's rho correlational analysis results for women.

**Women**	**Exercise**	**Mental distress**	**Breakfast**	**Whole grain**	**Dairy**	**Caffeine**	**Fruits**	**Nuts**	**HGI Food**	**Meat**	**DGLV**	**Beans**	**Fish**	**Fast food**	**MV**	**Fish oil**
Exercise	1.000	–0.201^**^	0.179^**^	0.178^**^	−0.003	–0.067^*^	0.212^**^	0.199^**^	–0.075^**^	0.000	0.319^**^	0.190^**^	0.061^*^	–0.204^**^	0.142^**^	0.019
Mental distress		1.000	–0.216^**^	–0.049^*^	0.058^*^	0.136^**^	−0.044	–0.063^*^	0.077^**^	−0.003	–0.145^**^	–0.065^*^	–0.051^*^	0.211^**^	–0.069^**^	−0.011
Break fast			1.000	0.221^**^	−0.002	0.021	0.163^**^	0.190^**^	0.020	−0.005	0.122^**^	0.110^**^	0.093^**^	–0.218^**^	0.059^*^	0.063^*^
Whole grain				1.000	0.167^**^	0.139^**^	0.311^**^	0.286^**^	0.040	−0.046	0.261^**^	0.258^**^	0.047	–0.081^**^	0.086^**^	0.033
Dairy					1.000	0.340^**^	0.204^**^	0.069^**^	0.077^**^	0.152^**^	−0.027	−0.026	0.014	0.109^**^	0.016	0.024
Caffeine						1.000	0.221^**^	0.169^**^	0.021	0.047	0.056^*^	0.072^**^	−0.002	0.089^**^	0.041	0.023
Fruits							1.000	0.443^**^	0.013	−0.039	0.312^**^	0.246^**^	0.056^*^	–0.124^**^	0.088^**^	−0.036
Nuts								1.000	0.029	–0.086^**^	0.352^**^	0.300^**^	0.109^**^	–0.148^**^	0.060^*^	0.050^*^
HGI food									1.000	0.082^**^	0.013	0.098^**^	−0.024	0.138^**^	–0.053^*^	−0.042
Meat										1.000	0.053^*^	–0.189^**^	0.132^**^	0.117^**^	−0.003	0.041
DGLV											1.000	0.352^**^	0.169^**^	–0.158^**^	0.089^**^	0.005
Beans												1.000	0.109^**^	–0.172^**^	0.055^*^	0.054^*^
Fish													1.000	–0.061^*^	0.009	0.052^*^
Fast foods														1.000	0.035	0.003
MV															1.000	0.232^**^
Fish oil																1.000

*DGLV, dark green leafy vegetables; HGI, high glycemic index; MV: multivitamin. Bold signifies statistical significance*.

* *P < 0.05*;

** *P < 0.01*.

## Discussion

The study revealed several interesting and novel findings that answer several questions in the literature. Each analytical approach used answered one piece of the puzzle. Our results confirmed the qualitative hypothetical SD model that following a healthy dietary pattern or dietary practice is more likely to promote exercise and improve mental well-being. It also confirmed previously published reports that mental distress is associated with unhealthy dietary patterns and loss of healthy dietary practices (1, 45). However, the study went beyond these concepts to uncover remarkable dynamics between several food groups, dietary practices, exercise, and mental distress, and suggested that no single “healthy diet” is associated with mental well-being. Rather, the significance of the food groups in the diet modulates mental status. In addition, these intertwined relationships suggest that some food groups may positively impact motivation to eat healthy and exercise, which in turn improve mental well-being. Interestingly, this dynamic relationship displayed a differential response among young men and women. Generally, exercise spawned a positive effect of food groups on mental well-being and, in few cases, minimized the negative impact of food groups on mental well-being. To our knowledge, this study is the first to report these integrated and noteworthy findings.

### The Cluster Analysis

The cluster analysis categorized the different variables into groups that share a high degree of association among young men and women. Interestingly, the clusters generated common features between the two genders, whereby it showed an association between dietary patterns, exercise frequency and mental well-being. Interestingly, the cluster analysis produced similar patterns between gender. Among young men, cluster 1 reflected a healthy dietary practice with frequent exercise and mental well-being. Cluster 2 described a healthy dietary pattern with frequent exercise and mental well-being, while cluster 3 revealed an unhealthy dietary pattern with lower frequency of exercise and mental well-being. Among women, cluster 1 represented an unhealthy dietary pattern, low exercise frequency and mental well-being, while cluster 2 revealed a healthy dietary pattern with more frequent exercise and mental well-being.

#### Observations From the Cluster Analysis

Results from the cluster analysis generated three significant observations: (1) there is an association between exercise and mental well-being regardless of the quality of the dietary pattern followed, (2) healthy dietary patterns and healthy dietary practices (eating breakfast, use of multivitamin or fish oil supplements) are associated with higher exercise frequency and, (3) unhealthy dietary patterns are associated with lower frequency of exercise. Taking all into consideration, healthy dietary or dietary practices may be promoting motivation to exercise, which eventually support mental well-being. These results support the CLD segments of the SD model.

### PCA

The next step was the use of PCA to produce a low-dimensional representation of the variables with the highest variance. The advantage of PCA is its ability to group variables into patterns while assigning a component loading that represents the weight of the variable in each pattern. This important feature added to the findings from the cluster analysis. PCA generated a similar pattern of principal components (PCs) for men and women. The 3 PCs identified for men and women represent a healthy dietary pattern, which included frequent exercise; an unhealthy dietary pattern, mental distress, and no exercise; and healthy dietary practices with frequent exercise and mental well-being. However, the results from PCA went beyond confirming some of the cluster analysis conclusions, by describing the potential role of certain food group variables on mental well-being.

#### Observations Form PCA

Interestingly, although PC1 in men and women (the healthy dietary pattern and exercise) included several food groups known to associate with mental well-being and exercise, no significant positive or negative loading surfaced for mental distress. In addition, PC1 included a strong loading for caffeine and excluded high-quality proteins such as dairy and meat. The latter are high in precursors for serotonin and dopamine, tryptophan, and tyrosine, respectively, which modulate brain chemistry. Nevertheless, it is worth noting that consumption of large quantities of animal protein is typically associated with higher saturated fat and cholesterol intake. Depending on the meat processing procedure, salt and other additives may become a concern as well (46).

The results from PC1 suggest that potential thresholds for exercise, caffeine and high-quality proteins may exist to reach mental well-being. In PC1, exercise loading was about double the caffeine weight. However, absence of high-quality proteins in a healthy dietary pattern may have disturbed the amino acid pool needed for optimal brain chemistry (47). As for PCs 2, the unhealthy dietary pattern was detected for both genders, as well, with similar observations. When caffeine weight is significant with exclusion of exercise, mental distress has a significant loading. This finding suggests a loss of a positive mediation effect of exercise on caffeine, which negatively impacted mental well-being. It also explained the finding from the cluster analysis that depicted mental well-being with an unhealthy dietary pattern and exercise, where caffeine was not a significant part of these patterns. A quick review of the literature failed to return any published report on the mediation effect of exercise on caffeine in relation to mental distress. The closest study was by Sarris et al. (48) who suggested that frequent exercise and reduction of caffeine may improve symptoms of anxiety.

Although high-quality proteins were consumed in PC 2, there was an evident absence of nutrient-dense food, which reinforces one segment of the CLD describing that a balanced healthy diet is needed to support motivation to exercise and promote mental-well-being. Thus, the cluster analysis and PCA for men and women supported this segment of the CLD hypothesis that a healthy dietary pattern or a healthy dietary practice is associated with exercise, which improves mental well-being.

#### Common Observations Generated From Cluster Analysis and PCA

Supplements use pattern along with eating breakfast, fish and minimal other healthy food choices may have a positive impact on mental distress when exercise is included. Interestingly, this was a common pattern observed between the cluster analysis and PCA that grouped breakfast, supplement use, exercise and eating fish. Breakfast has been reported to improve mental well-being through reduction of morning cortisol levels and improvement in blood glucose (49–51). It seems that multivitamin supplementation compensates for the micronutrients' flaws in the diet. In fact, deficiency in vitamins, namely B-complex and omega-3 has been correlated with mental health disorders (52, 53), which may explain the association between multivitamin and fish oil use, and fish eating with mental well-being. In addition, fish may provide the right balance between tryptophan and tyrosine needed for optimal brain chemistry, when caffeine is not consumed.

Cluster and PC analyses generated models of healthy and unhealthy dietary patterns; however, the different weights of food groups and exercise levels were associated differently with mental distress. This observation suggests that frequency of food group consumption (or a threshold) may have differential effects on mental health. Additionally, exercise may modify the threshold of the dietary factors on mental distress. A more constant observation noted was that the absence of caffeine and frequent exercise was associated with a positive mood, which supports the recommendations by Sarris et al. (48). Even when consuming a healthy diet, level of caffeine consumption may modulate the degree of mental distress. Combining the findings from the cluster analysis and PCA, few hypotheses emerged. Since unhealthy diets are associated with lower exercise frequency and mental distress, food groups may be neurochemically modulating the motivation to exercise. In fact, altering the optimal balance between the Large Neutral Amino Acids (LNAA), typically induced by a high protein diet (47, 54), severely impact mood by fluctuating brain serotonin and dopamine levels (47, 55). Additionally, a potential dose-dependent effect of caffeine may be negatively impacting mental well-being as well as motivation to exercise.

#### Caffeine and Mental Distress

Caffeine breakdown competes with sex hormones metabolism for the same cytochrome P450 1A2 enzyme (56). Caffeine is a stimulant and an antagonist of adenosine A_1_ and A_2A_ receptors; therefore, it is associated with risk of sleep problems, anxiety and mood disorders (57). In addition, caffeine modulates the hypothalamic-pituitary-adrenocortical (HPA) axis and elevates glucocorticoid levels (58, 59). Adrenal glucocorticoids stimulate phenylethanolamine N-methyltransferase (PNMT) that methylate norepinephrine to produce epinephrine in the adrenal medulla (60). Therefore, caffeine at high doses and with its delayed metabolism imposes a sustained caffeine's activity on PNMT, which increases the stress response. In return, stress modulates neural mechanisms associated with motivated behavior (61), which explains our observations.

However, there is a lack of research on the effect of individual food groups and caffeine on motivation or behavior. Consequently, there was a need to explain these observations and investigate the mediating effects of exercise on mental distress when these food groups are consumed. The mediation correlation analysis revealed the association between individual food groups, dietary practices, exercise, and mental distress. Although it supported the SD model hypothesis that diet, dietary practices and exercise are interconnected, it proposed a differential gender-based response between food groups, exercise and mental well-being. Exercise reduced the negative impact of triggers of mental distress and boosted the positive effects of food groups for both young men and women supporting the SD model hypothesis. Interestingly, the dietary triggers of mental distress were different between each gender.

### Spearman Correlation Analysis

Spearman correlation analysis confirmed the gender-based MA findings and revealed additional noteworthy conclusions. For women, exercise was associated with eating a spectrum of nutrient dense food (a healthy diet) and eating breakfast and taking multivitamin (healthy dietary practices). It was inversely associated with mental distress, caffeine intake, HGI food, and fast food. These findings support the CLD segment of the model that exercise boosts mental well-being which supports eating healthy and engaging in healthy dietary practices. This is in line with a previous report suggesting that women's mental well-being is associated with eating a spectrum of nutrient- dense food along with frequent exercise (31). Eating breakfast was strongly associated with taking multivitamin and fish oil supplements (healthy dietary practices), eating a spectrum of nutrient dense food (a healthy diet) and was inversely associated with mental distress and eating fast food. This finding supports the CLD segment that healthy dietary practices are likely to promote exercise, which in turn improves mental well-being and increases the motivation to eat healthy. All the components of a healthy diet (whole grain, fruits nuts, dark green leafy vegetables, beans, and fish) had shown an interrelationship, which means that eating one healthy food group is likely to encourage consumption of others. They were all positively associated with eating breakfast and supplement use (dietary practice), exercise, and avoiding fast food. Interestingly, all but fruits from the healthy food groups are inversely associated with mental distress.

As for men, there were similar and unexpected findings. Exercise was also strongly associated with eating a spectrum of healthy food and engaging in healthy dietary practices. Exercise was inversely associated with mental distress and eating fast food. These findings also support the CLD segment of the model that exercise boosts mental well-being, which supports eating healthy and engaging in healthy dietary practices. Eating breakfast was strongly associated with multivitamin supplements use, exercise, and a spectrum of nutrient dense food. It was negatively associated with mental distress. This finding supports the CLD segment that healthy dietary practices are likely to promote exercise, which in turn improves mental well-being and increases motivation to eat healthy. All the components of a healthy diet (whole grain, fruits nuts, dark green leafy vegetables, beans, and fish) had shown an interrelatedness, which implies that eating one healthy food group is likely to encourage consumption of others, as seen in women. All healthy food groups, except for nuts, are positively associated with eating breakfast and with some supplements use (dietary practice), exercise, and avoiding fast food. However, the unexpected finding was that none of the healthy food groups are inversely associated with mental distress. Mental distress in men was associated with high intake of caffeine and fast-food, lack of exercise and skipping breakfast. These findings are peculiar as they suggest that men's mental well-being is less reliant on a spectrum of nutrients as detected in women. Interestingly, this finding was previously reported by our research team through a distinct gender-based study that mental well-being in men is less likely to be associated with specific dietary patterns (31). A summary of all significant findings is provided in Figure 3B.

### Revisiting the CLD Segments of the SD Model

**Figure 4:**
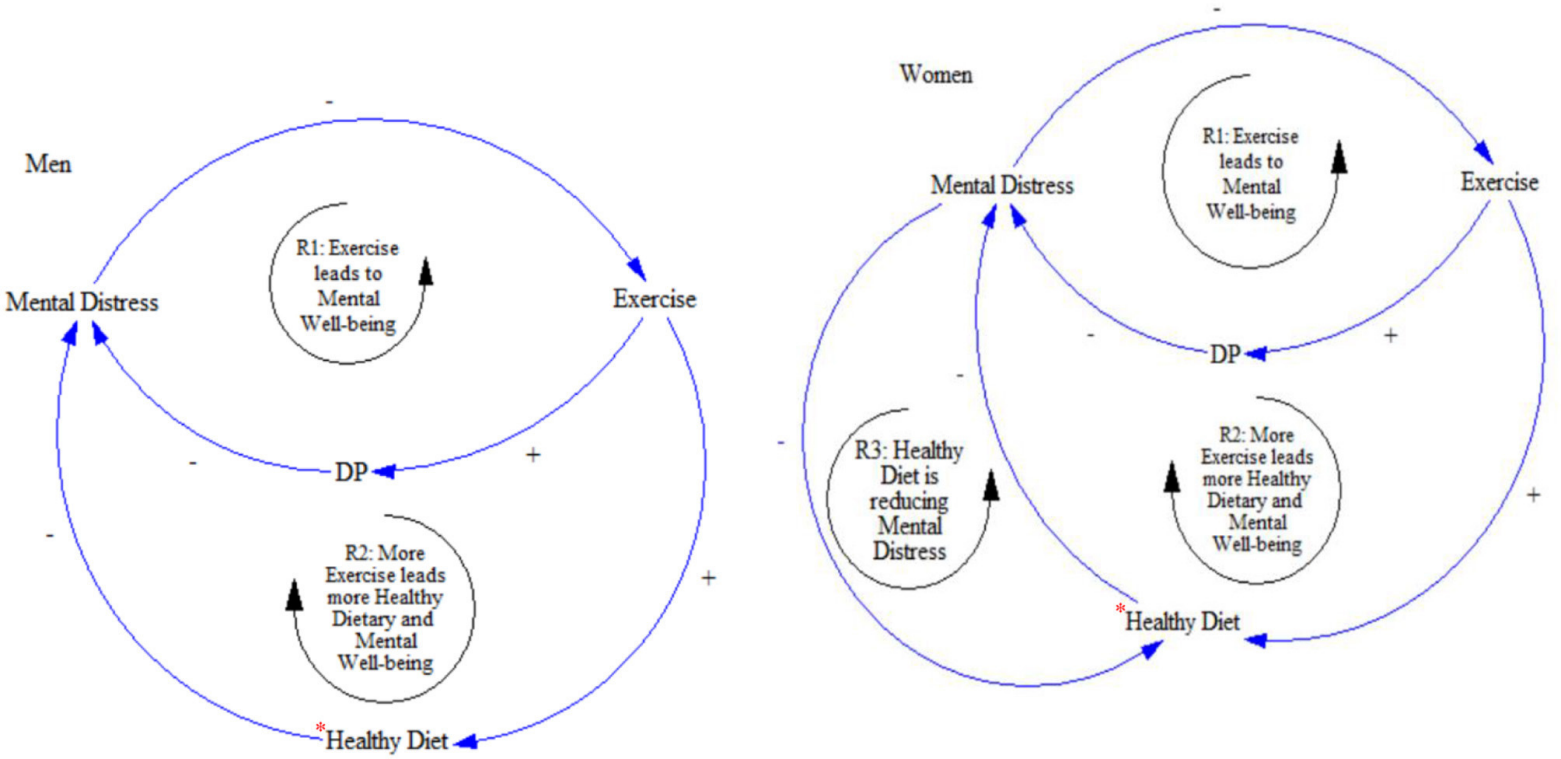
The revised CLD diagrams of the SD model based on gender. *Healthy diet is gender-based.

Our results necessitated a revision of the CLD segments based on gender since there is an obvious difference in the directional causality between diet and mental well-being among women. In the causal loop diagram, a positive causal link describes one factor promotes another and a negative explains how one factor inhibits another. A feedback loop is reinforcing (R) when one factor promotes another leading to further change in the same direction (Figure 4).

*a*. *Reinforcing loop 1: R1—Mental well-being due to exercise and dietary practices*. This loop was common to young men and women. Exercise encourages adaptation of healthy dietary practices, which eventually enhance mental well-being.
*b*. *Reinforcing loop 2: R2—Mental well-being due to exercise and a healthy diet*. Exercise is likely to improve diet quality, which improves mental well-being in both genders.
*c*. *Reinforcing loop 3: R3—Mental status is dependent on diet quality in young women*. Our analysis revealed that there is a positive effect from diet on mental status and a positive link from mental status to diet that is exclusive to women. In other words, a healthy diet supports mental well-being, and mental well-being leads to a healthy diet. Similarly, mental distress leads to a low-quality diet in young women.

### Strengths and Limitations of the Study

The major strengths of the study are the large sample size and the use of multiple robust analytical methods to extract the findings and validate the CLD segments of the hypothetical model. In addition, the study is reporting new findings that fill several gaps in the literature, namely that exercise significantly mediates the effect of food groups to promote mental well-being. Another interesting hypothesis generated from our results is that despite following a healthy diet and lifestyle, if triggers of mental distress exceed certain thresholds, mood is negatively impacted. Additionally, the results provide compelling evidence that mental health is modulated not only by a dietary pattern but by the weight of food groups and exercise frequency, which needs further investigation. Nevertheless, the limitations of this study include the convenience sample and its cross-sectional design, which limits the results to causal inference only Additionally, it does not take into consideration the differences in genetic, societal factors or any factor that may have affected the psychology of the individuals.

## Conclusion

Our results support the validation of the causal loop diagram by adopting a system dynamic modeling methodology, which may provide a framework for improving dietary and lifestyle factors based on gender. It could also serve as a context for research to further investigate frequency of food groups, dietary practices, and exercise and mental well-being. Although these factors behave commonly in young men and women, they may bear different thresholds that may modulate mental health. In other words, a differential gender-based repertoire may be needed to optimize diet quality, exercise, and mental well-being.

## Data Availability Statement

The original contributions presented in the study are included in the article/supplementary material, further inquiries can be directed to the corresponding author.

## Ethics Statement

The studies involving human participants were reviewed and approved by Internal Review Board at Binghamton University. The patients/participants provided their written informed consent to participate in this study.

## Author Contributions

LB: study conception and design, data interpretation, and manuscript writing. HN and DW: data collection and curation. DW: funding acquisition. HK: contribution to manuscript. HK and NS: data interpretation and statistical analyses, manuscript editing. All authors approved the final version.

## Conflict of Interest

The authors declare that the research was conducted in the absence of any commercial or financial relationships that could be construed as a potential conflict of interest.
